# Physical Therapists’ Attitudes, Beliefs, and Barriers Regarding Fall Screening and Prevention among Patients with Knee Osteoarthritis: A Cross-Sectional Study

**DOI:** 10.3390/healthcare12070718

**Published:** 2024-03-25

**Authors:** Mashael Alsobhi, Afnan Gmmash, Rawan Aldhabi, Muataz R. Almaddah, Alaa Ameen, Fae Almotairi, Reem Basuodan, Fayaz Khan

**Affiliations:** 1 Department of Physical Therapy, Faculty of Medical Rehabilitation Sciences, King Abdulaziz University, Jeddah 22252, Saudi Arabia; 2 Department of Rehabilitation Sciences, College of Health and Rehabilitation Sciences, Princess Nourah bint Abdulrahman University, P.O. Box 84428, Riyadh 11671, Saudi Arabia

**Keywords:** fall, knee, osteoarthritis, prevention, survey

## Abstract

Falls are commonly associated with knee osteoarthritis and represent a significant financial burden on the healthcare system. Therefore, the discovery of physical therapists’ attitudes and practices regarding fall screening and prevention among patients with osteoarthritis should be investigated. Moreover, this study aimed to identify barriers that might limit its implementation among this population. A cross-sectional study design was used to collect the data. The electronic survey targeted licensed physical therapy professionals who currently work in clinical or academic settings in Saudi Arabia. The data were analyzed descriptively and inferentially using chi-square. Two hundred and six licensed physical therapists completed the survey, 119 females (57.8%) and 87 males (42.2%). The results of the structural equation modelling analysis showed that intention to use fall screening and management strategies was positively associated with the history of falls, identifying risk factors of falls, and documentation of risk factors of falls (*p* ≤ 0.0001). The most reported barriers to implement fall screening and prevention were lack of knowledge (*n* = 92, 45%), lack of training/skills (*n* = 84, 41%), and time constraints (*n* = 57, 45%), followed by patient compliance with 38% of the responses. The findings highlighted the importance of identifying the key opportunities for knowledge translation in clinical practices to enhance the sufficient implementation of fall screening and management in osteoarthritis care.

## 1. Introduction

Knee osteoarthritis is a degenerative joint disease that involves the progressive loss of articular cartilage from wear and tear [1]. Knee osteoarthritis is a leading cause of disability globally, with a prevalence of 22.9% in individuals aged 40 years and older [2]. A recent study found a high prevalence of this disease in Saudi Arabia, with 24% to 60.9% of adults 60 years and above having knee osteoarthritis [3]. Numerous studies have confirmed that patients with knee osteoarthritis are at greater risk of falling than patients without knee osteoarthritis [4,5,6]. According to the World Health Organization (WHO), falling is defined as unintentionally resting on the ground or a lower surface [7]. It has been reported globally that 30% of older adults experience at least one fall or more annually [8]. Multiple factors may increase the risk of falls among knee osteoarthritis patients, and these can be divided into intrinsic and extrinsic risk factors [9]. Intrinsic risk factors can be subdivided into biomechanical, knee osteoarthritis symptoms, and the presence of comorbidities. Biomechanical factors include balance, strength, proprioception, and instability, while comorbidity risk factors include diabetes, fractures, and visual impairment. In regard to the extrinsic risk factor, assistive walking aids [10], slippery floors, inadequate lighting, loose rugs, unstable furniture, and obstructed walkways influence the prevalence of falls in the elderly [11]. Falls not only impact a patient’s physical capabilities but also their social and psychological conditions [7]. Additionally, injury from a fall can have a wide range of complications, such as fractures, tissue injuries, loss of confidence, fear of falling, or death. Adults with a history of falling develop a fear of future falls, which can negatively affect their confidence in their ability to perform activities and, subsequently, limit their quality of life [7]. Thus, falls and their associated injuries are a significant burden on both society and healthcare systems [12].

Many studies have investigated fall prevention programs for older adults [13,14,15]. To the best of our knowledge, no fall prevention programs have been developed specifically for patients diagnosed with knee osteoarthritis. Similarly, although patients with knee osteoarthritis are prone to falls [16], no fall prevention or fall risk screening recommendations are found in the international osteoarthritis clinical guidelines, such as those of the Osteoarthritis Society International (OARSI) and American College of Rheumatology [17,18]. The guidelines from these organizations emphasize weight reduction, pain management, exercise, and surgical referral as the primary management strategies for osteoarthritis [19,20,21,22,23,24,25,26]. Non-pharmacological treatment options, including physical therapy, are recommended by the American College of Rheumatology/Arthritis Foundation national guidelines as the first line of treatment for patients with osteoarthritis [27]. Older adults commonly experience both arthritis and falls. A study by Harris et al. [28] found that older adults with knee arthritis were more likely to experience recurrent falls regardless of the arthritis severity. While there is much research supporting the need for fall prevention in older adults, clinical care for arthritis generally provides less focus on this aspect of the treatment. Considering the present circumstances of a growing senior population and an increasing number of individuals with osteoarthritis, it is crucial to raise awareness and adopt appropriate treatments. However, there has been limited research undertaken to examine the present comprehension and methodologies employed by physical therapists concerning fall prevention in patients with knee osteoarthritis. It is crucial to have a comprehensive understanding of the disparities between the current evidence and clinical practice and to determine significant areas for improvement in healthcare.

Asiri et al. [29] conducted a study in Saudi Arabia documenting the knowledge of physicians, physical therapists, and nurses about fall risk factors and the practice of fall prevention among older adults in home healthcare settings. The researchers reported that physical therapists were least likely to report falls, after physicians and nurses. The study also showed that most of the physical therapists in the sample who worked in home healthcare could not identify the common risk factors for falls as identified in the literature, and approximately 14% of the physical therapists admitted to having little knowledge about fall risk. The study concluded that fall prevention training programs should be developed for physical therapists. Similarly, a study by Kalu et al. [30] found that the participating physical therapists were not familiar with WHO fall prevention strategies or any standardized fall prevention strategies [30]. Neither of these studies found a relationship between physical therapists’ knowledge, practice, and their demographic data, which included age, sex, experience, and work setting. It is important to mention that osteoarthritis comes in third place after stroke and cognitive dysfunction among health conditions that lead to falls. A study conducted in Australia by Ackerman et al. [19] showed that while most physical therapists had received training in the assessment of fall risk factors and fall prevention, many physical therapists indicated having moderate confidence in their ability to screen for fall risk factors and provide fall prevention programs to hip and knee osteoarthritis patients. The results of these studies are particularly relevant as it is well known that physical therapists commonly deal with osteoarthritis patients, which means they play a major role in providing patients with fall prevention strategies.

Thus, it is essential to investigate physical therapists’ perceptions of fall risk and prevention among patients with knee osteoarthritis. This study aimed to explore physical therapists’ understanding and attitudes toward fall risk screening and prevention programs for individuals with knee osteoarthritis based on multiple factors, including age, years of experience, subspecialty, working environment, and educational qualifications. The researchers also sought to determine the barriers that limit the implementation of fall risk screening and prevention programs among this population.

## 2. Materials and Methods

### 2.1. Participants

This cross-sectional study targeted licensed physical therapists who work in academic and non-academic settings and treat patients with knee osteoarthritis in Saudi Arabia. Therapists who did not treat any patients with knee osteoarthritis in the last three weeks were excluded from the study. The minimum sample size was determined using G-power 3.1 using effect size of 0.3 and α level of 0.05. A sample size of 143 was the minimum number of participants recommended to achieve a power of 80%. The convenience sampling method was used to recruit the participants.

### 2.2. Materials

A web-based survey was created using Google Forms. The online survey consisted of 23 questions that demonstrate the physical therapists’ perceptions and attitudes toward fall assessment and prevention provided for patients with knee osteoarthritis. The first part of the survey consisted of demographic questions (gender, age, country, educational level, specialty, years of experience, and the number of osteoarthritis patients treated in the last three weeks). In the second part of the survey, therapists were asked to rate their confidence in managing patients with knee osteoarthritis. The assessment of this question was determined using a 5-point Likert scale where 5 = extremely confident, 4 = quite confident, 3 = somewhat confident, 2 = slightly confident, and 1 = not confident at all. Then, they were asked to assign the level of importance of each risk factor for falls encompassed by patients with knee osteoarthritis based on their experience. The risk factors were listed in the survey based on previous studies [29,31], in addition to several questions regarding fall assessment and prevention strategies that physical therapists provide to their knee osteoarthritis patients. Part of the questions were adopted from research conducted in Australia [19] and Nigeria [30]. After formatting the questions, the survey was reviewed by five physical therapist experts for its clarity, order, and appropriateness, and the final edit was revised based on the experts’ comments to establish the face and content validity of the survey. To ensure face validity, the survey was given to five physical therapists who were familiar with the phenomena being studied and the survey methodology. The experts were asked to rate each item based on its face value and appropriateness using a Likert scale ranging from 1 (not important at all) to 5 (highly important). Then, the modified Delphi method was employed to establish content validity. A panel of five specialists was requested to rank each item based on its relativity, clarity, and appropriateness. Two rounds of expert revision were conducted to seek an 80% consensus on the survey questions’ validity. A full copy of the survey is available in Supplemental Materials.

### 2.3. Procedure

IRB approval was obtained from the Faculty of Medical Rehabilitation Sciences, King Abdulaziz University, Jeddah, Saudi Arabia (No. FMRS-EC-2022-013) before conducting the study. A self-administration anonymous survey was distributed on different social media platforms, including WhatsApp, Twitter, Telegram, Instagram, and Facebook. Information about the study, its purpose, its nature of confidentiality, and anonymity were posted clearly in the preface of the survey link. The survey started by asking the therapist if he/she is willing to voluntarily participate in the study. After obtaining the participation consent, respondents started to fill out the survey. The survey link was open from February to April 2022.

### 2.4. Statistical Analysis

All the data were reviewed for completeness. The data were entered into SPSS Statistics (Version 26). Descriptive statistics were calculated for all demographic variables using frequencies and percentages. Descriptive, exploratory data analysis methods helped to analyze the overall perception of physical therapists regarding fall management strategies among patients with knee osteoarthritis. Crosstabulation tests were used to describe the physical therapists’ opinions and attitudes toward falls among patients with knee osteoarthritis based on five factors including gender, subspecialty, employment sector, years of experience, and educational qualifications.

Furthermore, SPSS AMOS version 23 was used to perform structural equation modeling (SEM). The variables were grouped into 2 factors to find the significance of including screening of falls in patients with osteoarthritis: Factor 1 (assessment), which was grouped by history of falls, identifying risk factors of falls, and documentation of risk factors of falls, and factor 2 (management), which was grouped by planning to provide interventions to address risk factors of fall, providing treatment to address risk factors of fall, and educating patients on fall prevention strategies. The fit of the model was assessed using the following cut-off measures based on the literature of SEM. A significant χ^2^ and a comparative fit index (CFI) greater than 0.9, a chi-square fit statistic/degrees of freedom (CMIN/DF) less than 5, a goodness of fit index (GFI) greater than 0.9, and a root-mean square error of approximation (RMSEA) less than 0.08 was considered a good model fit [32]. The Consensus-Based Checklist for Reporting of Survey Studies (CROSS) was used to write the report for this study [33].

## 3. Results

### 3.1. Descriptive Statistics

A total of 206 physical therapy practitioners from different work settings filled out the electronic survey. Most of the participants (*n* = 98, 47.6.%) were between 25 to 34 years old. More than half, 119 (57.8%), of the participants were females while 87 (42.2%) were males. The majority of the participants (*n* = 174, 84.5%) were working in non-academic sectors such as hospitals and clinics. A large number of the participants (*n* = 148, 71.8%) were bachelor holders. Furthermore, 175 out of 206 (85.0%) of the participants reported having fewer than 10 years of clinical practice experience, and 59 (28.6%) of the participants specialized in the musculoskeletal disorders. Most of the physical therapists (*n* = 78, 47.1%) have seen at least 1 to 5 osteoarthritis patients in the last 3 weeks. Only 88 (42.7%) participants stated that they are quite confident in managing patients with knee osteoarthritis. Table 1 shows further details of the participants’ demographic data.

### 3.2. Opinions of Physical Therapists Regarding the Risk Factors for Falls

**Figure 1:**
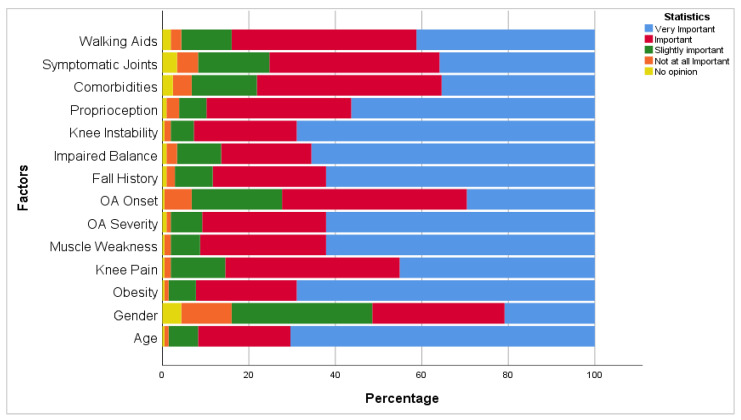
Physical therapists’ opinion about the importance of multiple factors for falls.

The results showed that 70.4% believe that age is a very important risk factor for falls among knee osteoarthritis individuals. Additionally, almost an equal percentage of physical therapist participants (68.9%) identified obesity and knee instability as very important factors. However, 11.7% thought that gender is not a risk identifier factor of fall among this population. The detailed overall physical therapists’ opinions toward multiple risk factors for falls among the knee osteoarthritis population are illustrated in Figure 1.

### 3.3. Management Options Provided to Patients with Knee Osteoarthritis

**Figure 2:**
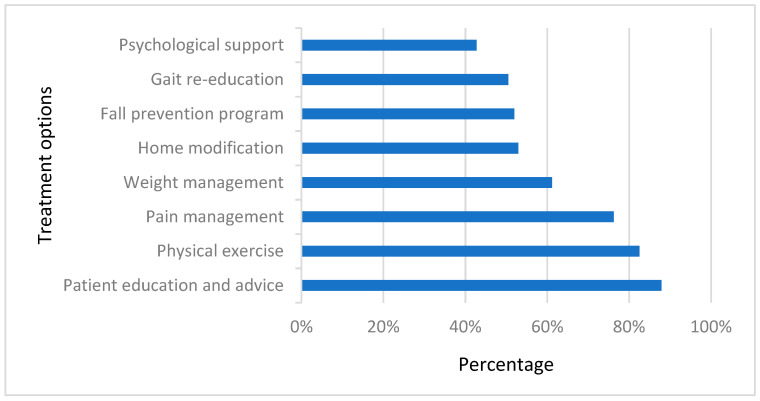
Frequently selected knee osteoarthritis treatment approaches. Patient education and exercises were the most reported treatment options used by physical therapists when treating knee osteoarthritis patients.

The most commonly reported type of management utilized by physical therapists was patient education (88%), followed by physical exercises (83%) and pain management (76%). A fall prevention program was chosen by 52% of the total sample as a treatment option for knee osteoarthritis cases. Figure 2 shows the percentages of different management approaches selected by physical therapists.

### 3.4. Common Mechanism of Falling

**Figure 3:**
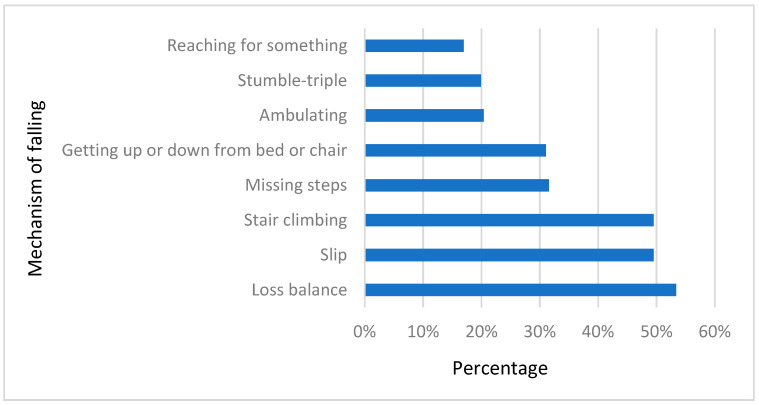
Physical therapists’ perceptions regarding the common cause of falling among knee osteoarthritis individuals.

Physical therapists were asked to indicate the common cause of falling among patients with knee osteoarthritis. Loss of balance (53%), stair climbing (50%), and slipping (50%) were the highest reported mechanisms of fall. However, 17% of the participants thought that reaching for something could cause people with knee osteoarthritis to fall. The percentages of each mechanism of falling are illustrated in Figure 3.

### 3.5. Analysis of Fall Screening and Prevention Attitudes by Physical Therapists’ Characteristics

A chi-square analysis was non-significant for the independent variables determined in the study. However, cross-tabulation analysis was used to describe the association between the variables.

#### 3.5.1. Current Practice of Physical Therapists

Results showed a detailed description of the physical therapists’ current practice in terms of identifying risk factor for falls, providing intervention specifically for falls, and educating patients about fall risk when managing patients with knee osteoarthritis based on a set of independent variables (gender, specialty, employment sector, experience, and education qualification).

##### Identify Risk Factors for Fall

Thirty-six male participants (41.4%) stated that they often identify fall risk indicators during their initial screening for patients with knee osteoarthritis compared to females. Based on specialization, both musculoskeletal and non-musculoskeletal physical therapists reported that identifying risk factors for falls is always checked during their initial assessment by 35.6% and 36.7% of the total sample, respectively. A large percentage of the participants (*n* = 66, 37.9%) who work in a non-academic sector always identify fall risk factors when assessing the osteoarthritis population. The majority of the participants (*n* = 66, 37.7%) who have over 10 years of clinical practice experience always identify fall risk factors.

##### Provide Intervention to Fall

Most of the male participants (*n* = 36, 41.4%) stated that they always provide fall interventions, while only two (2.3%) reported that they rarely provide it for knee osteoarthritis patients. For female participants, most of them (*n* = 41, 34.5%) often consider providing fall intervention to this population. Regarding the specialty, both musculoskeletal and non-musculoskeletal physical therapists were nearly equal in their opinion that fall intervention program is always essential for knee osteoarthritis patients (37.3%, 35.4%, respectively). Likewise, participants who work in both academic and non-academic settings revealed that they always design a fall intervention program for knee osteoarthritis patients (28.1% and 37.4%, respectively). Based on years of experience, participants with less or more than 10 years of experience had an approximately equal opinion on incorporating fall intervention in the treatment plan when managing patients with osteoarthritis (37.1% and 29.0% of the total sample, respectively). A large number of postgraduates (*n* = 20, 34.5%) expressed their opinion as they always plan for fall management. Similarly, 36.5% of participants with bachelor’s degree always employ fall intervention to minimize risk of fall among osteoarthritis individuals.

##### Educate Patient on Fall Prevention

An equal percentage of both genders reported that they always educate their patients about fall prevention strategies. Surprisingly, only 10 (31.3%) participants in the academic work setting stated that they always educate their patients about fall prevention. In comparison, 76 (43.7%) participants in non-academic settings stated that education always takes part in their treatment plan. Moreover, 25 (42.4%) participants who specialized in the musculoskeletal system reported that they always have to educate patients about risk factors for fall and the strategies to prevent it. The majority of the participants (*n* = 74, 42.3%) who had fewer than 10 years of experience stated that patient education is always incorporated into the treatment plan to prevent falls among the knee osteoarthritis population. Concerning education qualification, 64 (43.2%) of physical therapists with bachelor’s degrees stated that patient education about fall risk and prevention is always provided to the patients, while only 22 (37.9%) of the postgraduate holders always document patient education in the treatment plan for osteoarthritis patients. Table 2 summarizes the current practice for managing patients with osteoarthritis based on physical therapists’ sociodemographic factors.

#### 3.5.2. Fall Screening and Prevention

Most male and female participants with different specialties use fall screening and prevention in their clinical practice. Fall screening and fall prevention are mostly applied by employees in the non-academic sector (*n* = 124, 71.3%). Based on years of experience, 73.1% (*n* = 128) of participants who have fewer than 10 years of clinical practice experience applied fall screening and prevention with knee osteoarthritis patients. In addition, a large number of undergraduate participants (*n* = 109, 73.6%) reported using fall screening and prevention during the management process.

##### Awareness of Clinical Practice Guidelines (CPG) for Osteoarthritis

Table 3 shows the crosstabulation analysis based on a set of variables. For gender, very few male and female participants (32.6% and 27.7%, respectively) reported their agreement to be aware of CPG. For specialty, 96 (46.6%) of musculoskeletal physical therapists were not aware of any CPG that can be utilized when managing patients with osteoarthritis. The majority of the non-academic providers (*n* = 121, 69.9%) expressed their unfamiliarity with CPG. In addition, a high number of physical therapists with fewer than 10 years of experience (*n* = 122, 70.1%) did not express their awareness of CPG in clinical use. Based on education qualification, only 38 (25.7%) of undergraduate physical therapists were aware of CPG.

##### Training for Fall Prevention

The training for fall prevention, cross-tabulated with a set of explanatory variables, is shown in Table 3. Based on gender, nearly the majority of both male and female participants (80.5% and 84.9%, respectively) did not provide any training for fall prevention when treating patients with knee osteoarthritis. Very few physical therapists who specialized in the musculoskeletal system (*n* = 6, 10.2%) included fall prevention training in the osteoarthritis treatment protocol. For the employment sector, the majority of both non-academic and academic providers (81.3% and 83.3%, respectively) did not consider training for fall prevention with knee arthritis disorders. Most of the participants with fewer than 10 years of experience (*n* = 145, 83.4%) stated they do not train and educate their patients about fall prevention techniques. In terms of educational qualification, the majority of physical therapists who had bachelor’s degrees (*n* = 121, 81.8%) did not train patients with knee osteoarthritis on how to prevent falls. The details of fall screening and prevention are illustrated in Table 3.

**Table 3:** Training for fall prevention cross tabulated with a set of explanatory variables.

		Yes	No
**Fall Screening and Fall Prevention**			
Gender	Male	62 (71.3)	25 (28.7)
	Female	86 (72.3)	33 (27.7)
Specialty	Musculoskeletal	42 (71.2)	17 (28.8)
	Non-musculoskeletal	106 (72.1)	41 (27.9)
Employment sector	Academic	24 (75.0)	8 (25.0)
	Non-academic	124 (71.3)	50 (28.7)
Experience	Fewer than 10 years	128 (73.1)	47 (26.9)
	More than 10 years	20 (64.5)	11 (35.5)
Qualification	Postgraduate	39 (67.2)	19 (32.8)
	Undergraduate	109 (73.6)	39 (26.4)
**Awareness of CPG**			
Gender	Male	28 (32.6)	58 (67.4)
	Female	33 (27.7)	86 (72.3)
Specialty	Musculoskeletal	24 (41.4)	34 (58.6)
	None-musculoskeletal	37 (25.2)	110 (74.8)
Employment sector	Academic	9 (28.1)	23 (71.9)
	Non-academic	52 (30.1)	121 (69.9)
Experience	Fewer than 10 years	52 (29.9)	122 (70.1)
	More than 10 years	9 (29.0)	22 (71.0)
Qualification	Postgraduate	23 (40.4)	34 (59.6)
	Undergraduate	38 (25.7)	110 (74.3)
**Training Fall Prevention**			
Gender	Male	17 (19.5)	70 (80.5)
	Female	18 (15.1)	101 (84.9)
Specialty	Musculoskeletal	6 (10.2)	53 (89.8)
	None-musculoskeletal	29 (19.7)	118 (80.3)
Employment sector	Academic	6 (18.8)	26 (81.3)
	Non-academic	29 (16.7)	145 (83.3)
Experience	Fewer than 10 years	29 (16.6)	146 (83.4)
	More than 10 years	6 (19.4)	25 (80.6)
Qualification	Postgraduate	8 (13.8)	50 (86.2)
	Undergraduate	27 (18.2)	121 (81.8)

### 3.6. Structural Equation Modeling

**Figure 4:**
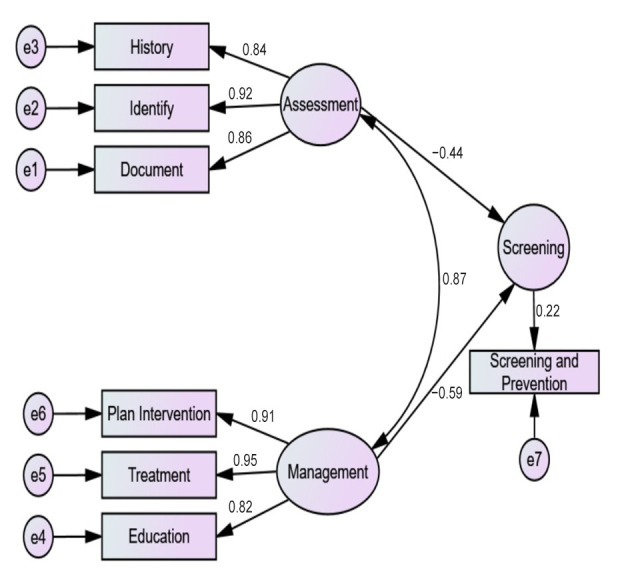
Path diagram of structural equation modeling predicting the factors associated with screening for falls in patients with osteoarthritis.

Figure 4 illustrates the path diagram of structural equation modeling predicting the factors associated with screening for falls in patients with osteoarthritis. The standardized regression weights between the history of falls and assessment were (Estimate; 0.8, *p* ≤ 0.0001), identifying risk factors of falls and assessment were (Estimate; 0.9, *p* ≤ 0.0001), and documentation of risk factors of falls and assessment were (Estimate; 0.8, *p* ≤ 0.0001). Similarly, for the management of the standardized regression weights between planning to provide interventions to address risk factors of falls and management were (Estimate; 0.9, *p* ≤ 0.0001), providing treatment to address risk factors of fall and management were (Estimate; 0.9, *p* ≤ 0.0001), and educating patients on fall prevention strategies and management were (Estimate; 0.8, *p* ≤ 0.0001). The goodness-of-fit (chi-square/degree of freedom (CMIN/DF) = 1.1, goodness-of-fit index (GFI) = 0.9, comparative fit index (CFI) = 0.9, root mean square error of approximation (RMSEA) = 0.0) explains a good model fit. The validity and reliability of the factors in the latent variable assessment were confirmed by the factor loadings in each (history; (0.9), identify; (0.9), document; (0.9)) with a Kaiser–Meyer–Olkin Measure of 0.7, *p* ≤ 0.0001 and for the factors in the latent variable management were confirmed by the factor loadings in each (plan intervention; (0.9), treatment; (0.9), education; (0.8)) with a Kaiser–Meyer–Olkin Measure of 0.7, *p* ≤ 0.0001. The model had an average CR value of 7.9; *p* ≤ 0.0001.

### 3.7. Barriers to Provide Fall Screening and Prevention

**Figure 5:**
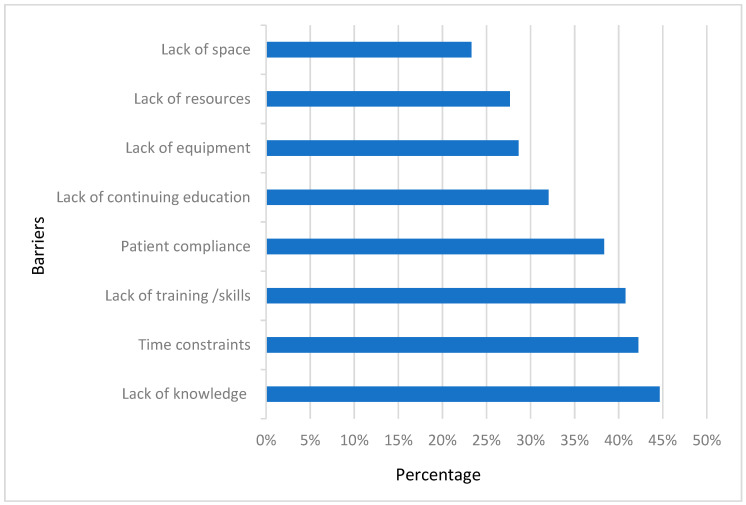
Barriers associated with implementing fall screening and prevention when managing knee osteoarthritis.

The most reported barriers were lack of knowledge (*n* = 92, 45%), lack of training/skills (*n* = 84, 41%), and time constraints (*n* = 57, 45%), followed by patient compliance with 38% of the responses. However, few participants (*n* = 48, 23%) perceived lack of space as a barrier that may limit performing fall screening and prevention strategies in clinical practices when treating patients with knee osteoarthritis. Figure 5 illustrates the barriers associated with implementing fall screening and prevention programs when managing the knee osteoarthritis population.

## 4. Discussion

This study explored physical therapists’ perceptions and attitudes regarding fall management among patients with knee osteoarthritis. It also investigated their beliefs regarding the importance of using fall screening and prevention in clinical practices in the context of multiple explanatory variables. Generally, this study found that physical therapists were somewhat aware of fall identification factors and management for this population in Saudi Arabia. Only 17.5% of the physical therapists described themselves as extremely confident when managing patients with knee osteoarthritis. Because falls are common among patients with knee osteoarthritis [9], physical therapists must be aware of common fall risks. In 2017, 50% of patients with knee osteoarthritis had a fall history [31]. According to this study’s results, most physical therapists can identify the common risk factors for falls described in the literature [10]. The results implied a limited integration of fall prevention into the clinical care of arthritis, so there is an urgent need for training tools on fall prevention that are specifically designed for clinicians who treat osteoarthritis populations, and that was similar to the findings from a study by Ackerman et al. [19] in Australia.

Furthermore, it was found that most physical therapists believed that age and obesity were the most important fall risk factors among patients with knee osteoarthritis. However, in this patient population, fall incidence is attributable to multiple factors, including muscle weakness, joint pain, joint stiffness, joint instability, impaired proprioception, fear of falling, and obesity [34,35]. In the current study, physical therapists reported that gender and the number of symptomatic joints were not important risk factors for falls among knee osteoarthritis patients. Conversely, Doré et al. [36] noted a strong relationship among fall risk, gender, and having multiple symptomatic joints. Physical therapy knowledge of the risk factors for falls among the osteoarthritis population must be further investigated.

Although this study’s findings revealed that physical therapists have a fair knowledge of factors that may lead to falls, prevention strategies were not the first treatment approach they preferred to implement with knee osteoarthritis patients. However, exercise, education, and pain management were the top treatment approaches indicated by the majority of physical therapists, which aligns with most international clinical guidelines for osteoarthritis management [17,18,37]. Fall prevention is not listed in those commonly used international guidelines for osteoarthritis, which might influence physical therapists’ decision-making in this study. A similar finding was found among Australian physical therapists, who professed suboptimal levels of confidence in fall management among the osteoarthritis population [19]. However, fall prevention is imperatively encouraged to be considered in osteoarthritis guidelines for optimal practice [9]. An osteoarthritis guideline update is required for consistency with the current evidence in the literature. A similar recommendation was supported by Rosadi et al. [9], who stated strong evidence to policymakers to emphasize fall prevention programs development to minimize falls among arthritis patients.

The literature contains limited studies investigating the common causes of falls, especially among individuals with knee osteoarthritis. However, tripping and slipping accounted for 60% of falls among individuals 65 years and older. Other factors were muscle weakness and loss of balance, accounting for 30.8% and 4.6% of falls, respectively. In this study, physical therapists indicated that loss of balance (53%), stair climbing (50%), and slipping (50%) were the top anticipated causes of falls among individuals with osteoarthritis, which is slightly similar to the documented causes of falls among healthy older adults. Tsonga et al. [38] reported that reaching was one of the major fall mechanisms among patients with severe knee osteoarthritis. However, reaching was the least common fall mechanism that physical therapists in this study identified. This study’s design did not allow for an in-depth understanding of physical therapists’ perceptions regarding the incidence of falls.

The standardized regression weight results showed good associations among the history of falls, identifying risk factors of falls, and documentation of risk factors of falls with physical therapists’ practices (assessment/management). However, the findings revealed that applying fall screening and prevention approaches was common among participants with fewer than 10 years of clinical experience. Conversely, a study found lower confidence in assessing falls risk and delivering fall prevention care for new graduates [19]. This variance might be due to differences in physical therapists’ practice patterns in Saudi Arabia. A deeper understanding of real-world physical therapy practices regarding osteoarthritis care is required. This could be acquired by conducting semi-structured interviews with physical therapists in the future.

The results of the path diagram of SEM showed a good model fit. Factors such as a history of falls, identifying fall risk factors, and documentation of fall risk factors were positively associated with both the screening and management of falls (*p* ≤ 0.0001). In the literature, physical therapists were the lowest of healthcare specialties in documenting a history of falls and identifying and documenting fall risk factors, especially compared to nurses and physicians [29]. Physical therapists need to address fall histories and identify and document fall risk factors when providing care to individuals with osteoarthritis. Physical therapists have the ability to design a plan that aims to decrease the likelihood of falling while also considering the patients’ specific goals and functional capabilities [39].

This study revealed the barriers physical therapists perceived regarding the limited implementation of fall prevention strategies when managing patients with knee osteoarthritis. The participants reported a lack of knowledge (45%), time constraints (42%), and a lack of training/skills (41%) as the major barriers to adopting fall practices. The results of this preliminary study emphasized the implementation of knowledge translation strategies in PT clinical practices in Saudi Arabia. Integrating methods that enrich physical therapists’ practice skills through audits, feedback, reminders, or training is encouraged. This study’s findings also offer insight into updating osteoarthritis guidelines and recommend adding fall screening and prevention.

The limitations of this study are primarily the self-reported survey and the convenience sampling method. The survey responses are susceptible to biases that may affect the validity of the findings. Recruiting the sample on social media platforms may limit the generalizability of the study results to the entire population of physical therapists in Saudi Arabia. This study also only recruited physical therapists, which could limit understanding of osteoarthritis practice; however, future studies might include other healthcare providers, such as physicians and nurses. This study was limited to one geographical region, so multi-site studies are recommended to be implemented by incorporating other Middle Eastern countries. Finally, the study sample was mainly based on physical therapists who are 35 years and younger with fewer years of experience; therefore, the findings might not reflect the real level of knowledge of adherence to fall screening and prevention strategies in osteoarthritis care.

## 5. Conclusions

The findings of the study revealed the practices of PTs that may contribute to the taking of appropriate actions in the future to make physical therapists’ practices and perceptions match those of the ongoing research. Accordingly, physical therapists should be encouraged to adopt fall risk screening and prevention programs in their clinical practices. The results of this study emphasized the need to include fall risk assessment and prevention programs for patients with knee osteoarthritis in clinical guidelines. Such guidelines would supply physical therapists with evidence-based methods for providing quality service to their patients. Physical therapists are required to be knowledgeable and confident about fall management since they play an important role in improving balance and preventing falls among older adults. Fall prevention should be addressed in PT clinical practices with osteoarthritis care to optimize the patients’ quality of care. This study suggested that the main barriers for not including fall screening and prevention strategies in clinical practice were lack of knowledge and skills or training, so training programs could be an effective approach that increases physical therapists’ knowledge and confidence in this area.

## Figures and Tables

**Table 1:** Demographic Characteristics of the Participants, Number of osteoarthritis Patients, and Confidence Level to Manage osteoarthritis Patients (*n* = 206).

Characteristics		Frequency (*n*)	Percentage (%)
Age (years)	Less than 25	65	(31.6)
	25–34	98	(47.6)
	35–44	29	(14.1)
	45–54	12	(5.8)
	55 and above	2	(1.0)
Gender	Male	87	(42.2)
	Female	119	(57.8)
Employment sector	Academic	32	(15.5)
	Non-academic	174	(84.5)
Education qualification	Undergraduate	148	(71.8)
	Postgraduate	58	(28.2)
Subspecialty	Musculoskeletal	59	(28.6)
	Non-Musculoskeletal	147	(71.4)
Experience	Fewer than 10 years	175	(85.0)
	More than 10 years	31	(15.0)
Number of patients in 3 weeks	None	47	(22.8)
	1–5	97	(47.1)
	6–10	42	(20.4)
	11–15	11	(5.3)
	16 or more	9	(4.4)
Confidence in treating osteoarthritis patients	Not confidence	11	(5.3)
	Slightly confident	29	(14.1)
	Somewhat confident	42	(20.4)
	Quite confident	88	(42.7)
	Extremely confident	36	(17.5)

**Table 2:** Distribution of physical therapists’ current practice to prevent falling, by confidence level in managing patients with knee osteoarthritis (*n* = 206).

		Always	Often	Sometimes	Rare	Never
**Identify risk factors for fall**						
Gender	Male	32 (36.8)	36 (41.4)	11 (12.6)	6 (6.9)	2 (2.3)
	Female	43 (36.1)	37 (31.1)	26 (21.8)	5 (4.2)	8 (6.7)
Specialty	Musculoskeletal	21 (35.6)	16 (27.1)	9 (15.3)	5 (8.5)	8 (13.6)
	Non-musculoskeletal	54 (36.7)	57 (38.8)	28 (19.0)	6 (4.1)	2 (1.4)
Employment sector	Academic	9 (28.1)	9 (28.1)	6 (18.8)	2 (6.3)	6 (18.8)
	Non-academic	66 (37.9)	64 (36.8)	31 (17.8)	9 (5.2)	4 (2.3)
Experience	Fewer than 10 years	66 (37.7)	63 (36.0)	31 (17.7)	8 (4.6)	7 (4.0)
	More than 10 years	9 (29.0)	10 (32.3)	6 (19.4)	3 (9.7)	3 (9.7)
Qualification	Postgraduate	18 (31.0)	17 (29.3)	10 (17.2)	5 (8.6)	8 (13.8)
	Undergraduate	57 (38.5)	56 (37.8)	27 (18.2)	6 (4.1)	2 (1.4)
**Provide intervention to Fall**						
Gender	Male	36 (41.4)	27 (31.0)	16 (18.4)	6 (6.9)	2 (2.3)
	Female	38 (31.9)	41 (34.5)	25 (21.0)	8 (6.7)	7 (5.9)
Specialty	Musculoskeletal	22 (37.3)	15 (25.4)	9 (15.3)	7 (11.9)	6 (10.2)
	Non-musculoskeletal	52 (35.4)	53 (36.1)	32 (21.8)	7 (4.8)	3 (2.0)
Employment sector	Academic	9 (28.1)	10 (31.3)	5 (15.6)	3 (9.4)	5 (15.6)
	Non-academic	65 (37.4)	58 (33.3)	36 (20.7)	11 (6.3)	4 (2.3)
Experience	Fewer than 10 years	65 (37.1)	58 (33.1)	37 (21.1)	11 (6.3)	4 (2.3)
	More than 10 years	9 (29.0)	10 (32.3)	4 (12.9)	3 (9.7)	5 (16.1)
Qualification	Postgraduate	20 (34.5)	18 (31.0)	6 (10.3)	6 (10.3)	8 (13.8)
	Undergraduate	54 (36.5)	50 (33.8)	35 (23.6)	8 (5.4)	1 (0.7)
**Educate Patient on Fall Prevention**						
Gender	Male	42 (48.3)	25 (28.7)	11 (12.6)	5 (5.7)	4 (4.6)
	Female	44 (37.0)	39 (32.8)	22 (18.5)	6 (5.0)	8 (6.7)
Employment sector	Academic	10 (31.3)	9 (28.1)	5 (15.6)	3 (9.4)	5 (15.6)
	Non-academic	76 (43.7)	55 (31.6)	28 (16.1)	8 (4.6)	7 (4.0)
Specialty	Musculoskeletal	25 (42.4)	16 (27.1)	6 (10.2)	5 (8.5)	7 (11.9)
	Non-musculoskeletal	61 (41.5)	48 (32.7)	27 (18.4)	6 (4.1)	5 (3.4)
Experience	Fewer than 10 years	74 (42.3)	54 (30.9)	31 (17.7)	10 (5.7)	6 (3.4)
	more than 10 years	12 (38.7)	10 (32.3)	2 (6.5)	1 (3.2)	6 (19.4)
Qualification	Postgraduate	22 (37.9)	16 (27.6)	7 (12.1)	5 (8.6)	8 (13.8)
	Undergraduate	64 (43.2)	48 (32.4)	26 (17.6)	6 (4.1)	4 (2.7)

## Data Availability

The data are available upon reasonable request.

## Supplementary Materials

The following supporting information can be downloaded at: https://www.mdpi.com/article/10.3390/healthcare12070718/s1.
