# Supplementary material for: Physical Therapists’ Attitudes, Beliefs, and Barriers Regarding Fall Screening and Prevention among Patients with Knee Osteoarthritis: A Cross-Sectional Study
Source: Healthcare (Basel). 2024 Mar 25;12(7):718. doi: 10.3390/healthcare12070718 (PMC11011968; doi:10.3390/healthcare12070718)
Supplement: Supplementary file 1 [file healthcare-12-00718-s001.zip › healthcare-2894520-supplementary.pdf]

Exploring Physical Therapists' Perceptions Regarding Risk Factors for falls among Patients With Knee Osteoarthritis

The importance of this study is to explore the knowledge and attitudes towards falls among patients with knee OA . You may exit the survey at any time. All information will be treated with confidentiality and no personal information will be disclosed to any one other than the research team. It should take less than **10 minutes** of your valuable time.

Thank you for your time
If you have any questions or concerns please feel free to contact the PI
Dr. Mashael Alsobhi   Email : mgalsobhi@kau.edu.sa

* Required

1. Are you willing to participate in the study? *

   Mark only one oval.

   - Yes
   - No

The following questions are designed to help the researchers analyze the results according to certain demographics. All information will remain confidential.

2. What is your gender? *

   Mark only one oval.

   - Male
   - Female

3. How old are you? *

Mark only one oval.

- <25
- 25-34 years
- 35-44 years
- 45-54 years
- 55 and older

4. Are you a physical therapy professional? *

Mark only one oval.

- Yes
- No

5. Are you a licensed physical therapist?

Mark only one oval.

- Yes
- No

6. In which country do you work as a physical therapist? *

Mark only one oval.

- Saudi Arabia
- United Arab Emirates
- Kuwait
- Oman
- Bahrain
- Qatar
- Other: ____________________

7. What is your highest educational qualification? *

Mark only one oval.

- Diploma
- Bachelor’s
- Master
- PhD
- DPT

8. What is your subspecialty? *

Mark only one oval.

- Orthopedic and musculoskeletal
- Sport injuries
- Cardiopulmonary
- Geriatric
- Vestibular
- Neurology
- General
- Other: ____________________

9. How many years of clinical practice experience do you have? *

Mark only one oval.

- Less than a year
- 1-5 years
- 6-10 years
- More than 10 years

10. In which setting, do you mainly work? *

Mark only one oval.

- Governmental hospital
- Private hospital
- Private clinic
- Academic
- Other: ____________________

11. On average, how many patients with knee OA have you seen in the past three weeks? *

Mark only one oval.

- 0
- 1-5
- 6-10
- 11-15
- 16 or more

12. How confident are you in managing patients with knee OA? *

Mark only one oval.

- Not confident at all
- Slightly confident
- Somewhat confident
- Quite confident
- Extremely confident

13. Based on your clinical experience, assign the level of importance to each of the following risk factors for falls among knee OA patients. *

Mark only one oval per row.

	Very important	Important	Slightly important	Not at all Important	No opinion
**Age**	⬭	⬭	⬭	⬭	⬭
**Gender**	⬭	⬭	⬭	⬭	⬭
**Obesity**	⬭	⬭	⬭	⬭	⬭
**Knee pain**	⬭	⬭	⬭	⬭	⬭
**Muscle weakness**	⬭	⬭	⬭	⬭	⬭
**OA severity**	⬭	⬭	⬭	⬭	⬭
**OA onset**	⬭	⬭	⬭	⬭	⬭
**Previous fall history**	⬭	⬭	⬭	⬭	⬭
**Impaired balance**	⬭	⬭	⬭	⬭	⬭
**Knee instability**	⬭	⬭	⬭	⬭	⬭
**Impaired proprioception**	⬭	⬭	⬭	⬭	⬭
**Comorbidities presence**	⬭	⬭	⬭	⬭	⬭
**↑number of symptomatic joints**	⬭	⬭	⬭	⬭	⬭
**Walking aids use**	⬭	⬭	⬭	⬭	⬭

14. Which of the following mechanisms of falls do you think are common among patients with knee OA? (Select all that apply) *

Check all that apply.

- [ ] Stumble-triple
- [ ] Slip
- [ ] Loss of balance
- [ ] Missing steps
- [ ] Ambulating
- [ ] Stair climbing
- [ ] Reaching for something
- [ ] Getting up or down from bed or chair
- [ ] Other: ______________________

15. Are you familiar with any clinical practice guidelines of knee OA? *

Mark only one oval.

- ( ) Yes
- ( ) No
- ( ) Not sure
- ( ) Other: ______________________

16. If your previous answer was yes, please write your clinical practice guidelines that you use

____

17. Do you think it is important to include fall screening and fall prevention in clinical practices when managing patients with knee OA? *

Mark only one oval.

- Yes
- No
- Not sure

18. If your previous answer was yes, which of the following fall assessment tool do you frequently utilize with your knee OA patients? (Select all that apply) *

Check all that apply.

- [ ] Falls Efficacy Scale International (FES-I)
- [ ] Activities-specific Balance Confidence (ABC) Scale
- [ ] Morse Fall Scale (MFS)
- [ ] Timed Up-and-Go (Tug)
- [ ] Fall Risk Assessment Tool (FRAT)
- [ ] None
- [ ] Other: ______________________

19. Which of the following management do you usually provide for patients with knee OA?(Select all that apply) *

Check all that apply.

- [ ] Patient education and advice
- [ ] Weight management
- [ ] Home modifications
- [ ] Gait re-education
- [ ] Pain management
- [ ] Physical exercise
- [ ] Fall prevention program
- [ ] Psychological support
- [ ] Other: ______________________

20. The following questions relate to your current practice as a Physical Therapist. Please indicate your frequency utilization of the following when treating patients with knee OA: *

Mark only one oval per row.

	Always	Often	Sometimes	Rare	Never
**In your initial assessment of patients, how often do you ask if they have a history of falls?**	◯	◯	◯	◯	◯
**In your initial assessment of patients, how often do you identify risk factors for falling?**	◯	◯	◯	◯	◯
**In your assessment of patients, how often do you document risk factors for falling?**	◯	◯	◯	◯	◯
**In your assessment planning, how often do you provide interventions to address fall risk factors?**	◯	◯	◯	◯	◯
**In your treatment plan, how often do you provide interventions to address fall risk factors?**	◯	◯	◯	◯	◯
**In your treatment plan, how often do you educate OA patients on fall prevention strategy?**	◯	◯	◯	◯	◯

21. Based on your clinical experience, what do you perceive as the barriers associated with implementing fall screening and prevention in clinical practice when managing patients with knee OA? (Select all that apply) *

Check all that apply.

- [ ] Time constraints/competing clinical priorities
- [ ] Lack of knweldge
- [ ] Lack of training /skills
- [ ] Lack of resources
- [ ] Lack of continuing education
- [ ] Lack of equipment
- [ ] Lack of space
- [ ] Patient compliance
- [ ] Other: ________________

22. Have you received any specific training for falls prevention programs/interventions? *

Mark only one oval.

- ◯ Yes
- ◯ No

23. If your previous answer was yes, Please mention the type/name of training you have received

____

This content is neither created nor endorsed by Google.

Google Forms
